# The VMAT-2 Inhibitor Tetrabenazine Affects Effort-Related Decision Making in a Progressive Ratio/Chow Feeding Choice Task: Reversal with Antidepressant Drugs

**DOI:** 10.1371/journal.pone.0099320

**Published:** 2014-06-17

**Authors:** Patrick A. Randall, Christie A. Lee, Eric J. Nunes, Samantha E. Yohn, Victoria Nowak, Bilal Khan, Priya Shah, Saagar Pandit, V. Kiran Vemuri, Alex Makriyannis, Younis Baqi, Christa E. Müller, Merce Correa, John D. Salamone

**Affiliations:** 1 Department of Psychology, University of Connecticut, Storrs, Connecticut, United States of America; 2 Center for Drug Discovery, Northeastern University, Boston, Massachusetts, United States of America; 3 Pharma-Zentrum Bonn, Pharmazeutisches Institut, Pharmazeutische Chemie, Universität Bonn, Bonn, Germany; 4 Department of Chemistry, Faculty of Science, Sultan Qaboos University, Muscat, Oman; 5 Àrea de Psicobiologia, Campus de Riu Sec, Universitat Jaume I, Castelló, Spain; University of Chicago, United States of America

## Abstract

Behavioral activation is a fundamental feature of motivation, and organisms frequently make effort-related decisions based upon evaluations of reinforcement value and response costs. Furthermore, people with major depression and other disorders often show anergia, psychomotor retardation, fatigue, and alterations in effort-related decision making. Tasks measuring effort-based decision making can be used as animal models of the motivational symptoms of depression, and the present studies characterized the effort-related effects of the vesicular monoamine transport (VMAT-2) inhibitor tetrabenazine. Tetrabenazine induces depressive symptoms in humans, and also preferentially depletes dopamine (DA). Rats were assessed using a concurrent progressive ratio (PROG)/chow feeding task, in which they can either lever press on a PROG schedule for preferred high-carbohydrate food, or approach and consume a less-preferred lab chow that is freely available in the chamber. Previous work has shown that the DA antagonist haloperidol reduced PROG work output on this task, but did not reduce chow intake, effects that differed substantially from those of reinforcer devaluation or appetite suppressant drugs. The present work demonstrated that tetrabenazine produced an effort-related shift in responding on the PROG/chow procedure, reducing lever presses, highest ratio achieved and time spent responding, but not reducing chow intake. Similar effects were produced by administration of the subtype selective DA antagonists ecopipam (D1) and eticlopride (D2), but not by the cannabinoid CB1 receptor neutral antagonist and putative appetite suppressant AM 4413, which suppressed both lever pressing and chow intake. The adenosine A_2A_ antagonist MSX-3, the antidepressant and catecholamine uptake inhibitor bupropion, and the MAO-B inhibitor deprenyl, all reversed the impairments induced by tetrabenazine. This work demonstrates the potential utility of the PROG/chow procedure as a rodent model of the effort-related deficits observed in depressed patients.

## Introduction

Motivation is a complex process that involves multiple behavioral functions and neural circuits [1]–[4]. Organisms are directed towards or away from stimuli, they can respond to primary motivational stimuli and conditioned cues, and under some conditions they can demonstrate high levels of behavioral activation [2], [5]–[8]. One of the manifestations of activational aspects of motivation is that organisms can show robust activity in the initiation and maintenance of motivated behavior, leading to substantial and persistent work output in their instrumental (i.e., reinforcer-seeking) actions. Thus, organisms can overcome response costs separating them from motivational stimuli, and frequently they must make effort-related decisions based upon cost/benefit analyses [1], [2]. In the last few years, there has been growing interest in the neural circuitry underlying effort-based processes, both in animals [2], [5], [9]–[15] and humans [16]–[20]. Forebrain circuits regulating exertion of effort and effort-related choice behavior involve several structures, including basolateral amygdala and prefrontal/anterior cingulate cortex [10], [14], [21], ventral pallidum [13], [22], and nucleus accumbens [5], [15], [23]–[26].

Effort-based decision-making is generally studied using tasks that offer a choice between high effort instrumental actions leading to more highly valued reinforcers vs. low effort options leading to less valued reinforcers. In animal studies, such tasks include a T-maze task that uses a vertical barrier to provide the effort-related challenge [23], [26], [27], [28], effort discounting tasks [9], [12], [29], and operant behavior procedures that offer animals a choice between responding on ratio schedules for preferred reinforcers vs. approaching and consuming a less preferred food [1], [30], [32]. Several studies in this area have focused on the effort-related effects of brain dopamine (DA) systems, particularly accumbens DA. Across multiple tasks, low doses of DA antagonists and accumbens DA depletions or antagonism shift choice behavior by decreasing selection of the high effort/high reward option and increasing selection of the low effort/low reward choice [5], [9], [23], [26], [33]. The effects of DAergic manipulations on effort-based allocation of responding are not explained by changes in appetite, food consumption or preference, or discrimination of reward magnitude [23], [30]–[32], [34], [35]. Furthermore, the effort-related effects of DA antagonism can be reversed by co-administration of adenosine A_2A_ antagonists such as istradefylline, MSX-3 and MSX-4 [25], [27], [36]–[40].

It has been suggested that tasks measuring effort-based decision making could be used to model the effort-related motivational symptoms of depression and other disorders [5], [15], [41]–[43]. People with depression and related disorders not only display alterations in mood or affect, but also can show profound psychomotor/motivational impairments (e.g. lassitude, anergia, fatigue, psychomotor retardation; [5], [44]–[46]). Tests of effort-related decision making have been developed in humans [47], and recent studies have shown that people with major depression show reduced selection of high effort alternatives [48]. The present work investigated the effort-related effects of tetrabenazine (TBZ), which is an inhibitor of VMAT-2 (vesicular monoamine transporter- type 2). By inhibiting VMAT-2, TBZ blocks vesicular storage and depletes monoamines, with its greatest impact being upon striatal DA [49], [50]. TBZ is used to treat Huntington's disease, but major side effects include depressive symptoms, including fatigue [51]–[53]. TBZ has frequently been used in studies involving animal models of depression [54]–[56], and the present studies assessed the effects of TBZ on performance of a concurrent progressive ratio (PROG)/chow feeding choice task [32]. With this task, rats have the choice of lever pressing on a PROG schedule reinforced by preferred high-carbohydrate pellets vs. approaching and consuming a less preferred laboratory chow. This choice procedure is useful because the PROG schedule requires that the animal repeatedly makes within-session choices between lever pressing and chow intake under conditions in which the ratio requirement is gradually increasing. Previous work with this task demonstrated that the DA D2 family antagonist haloperidol suppressed PROG lever pressing, highest ratio achieved, and time spent responding, but did not suppress chow intake [32]. These effects of DA antagonism differed substantially from administration of the cannabinoid CB1 inverse agonist and putative appetite suppressant AM251, and also differed from the effects of reinforcer devaluation by pre-feeding, both of which substantially suppressed chow intake under conditions that also reduced lever pressing [32]. As well as studying the effects of TBZ in the present work, additional drugs were administered so their effects could be compared with TBZ (the D1 antagonist ecopipam, the D2 antagonist eticlopride, and the CB1 receptor neutral antagonist AM4113). Finally, several putative and established antidepressant drugs (the adenosine A_2A_ antagonist MSX-3, the catecholamine uptake blocker bupropion, the MAO-B inhibitor deprenyl, and the COMT inhibitor tolcapone) were assessed for their ability to reverse the effects of TBZ. This work was conducted in order to work towards the development of animal models of the effort-related motivational symptoms of depression and other disorders [5], [41], [43].

## Materials and Methods

### Animals

Ninety four adult male Sprague-Dawley rats (Harlan, Indianapolis, IN, USA) were housed in a colony at 23 °C with 12-h light/dark cycles (lights on at 0:700 h). Rats weighed 300–350 g at the beginning of the study, and were initially food deprived to 85% of their free-feeding body weight for training. Rats were fed supplemental chow to maintain weight throughout the study, with water available ad libitum in the home cages. Despite food restriction, rats were allowed modest weight gain (approximately 10%) throughout the experiment, to be consistent with the normal growth of the animals. All animal protocols were approved by the University of Connecticut Institutional Animal Care and Use Committee, and followed NIH guidelines.

### Pharmacological Agents and Dose Selection

The VMAT-2 inhibitor tetrabenazine (Tocris Bioscience) was dissolved in a solution of 20% dimethylsulfoxide (DMSO) and saline and then titrated with 1N HCl and sonicated until dissolved. A pH matched vehicle solution of 20% DMSO and saline was used for the vehicle control for TBZ. The DA D1 antagonist ecopipam and the DA D2 antagonist eticlopride (Tocris Bioscience) were dissolved in 0.9% saline, which also served as the vehicle control for these studies. The adenosine A_2A_ antagonist MSX-3 was synthesized in the laboratory of Christa Müller (University of Bonn, Bonn, Germany) and was dissolved in 0.9% saline solution and then pH adjusted with 1N NaOH to a final pH of 7.4. Saline served as the vehicle control for MSX-3. The catecholamine uptake inhibitor bupropion hydrochloride (Alfa Aesar, Ward Hill, MA) and the MAO-B inhibitor deprenyl hydrochloride (Tocris Bioscience) were dissolved in 0.9% saline, which also served as the vehicle control for these studies. The catechol *O*-methyl transferase (COMT) inhibitor tolcapone (Toronto Research Chemicals, Toronto Canada) was dissolved in 0.9% saline solution and then titrated with Tween 80 and sonicated until dissolved. Saline was used as the vehicle control for tolcapone. AM4113 was synthesized in the laboratory of Alex Makriyannis (Center for Drug Discovery, Northeastern University, Boston, MA), and was dissolved in DMSO, Tween 80, and 0.9% saline at a ratio of 1∶1∶8. All doses were selected based on previous work [32], [35], [37], [39], [57] or from unpublished preliminary studies from this lab. All drugs were administered intraperitoneally (IP).

### Behavioral Procedures

Behavioral sessions were conducted in operant conditioning chambers (28×23×23 cm^3^; Med Associates). Rats were initially trained to lever press on a continuous reinforcement schedule (30-min sessions; 45-mg pellets, Bioserve, Frenchtown, NJ, USA) for 1 week, and then were shifted to the PROG schedule (30-min sessions, 5 days/week) for several additional weeks. For PROG sessions, the ratio started at FR1 and was increased by one additional response every time 15 reinforcers were obtained (FR1×15, FR2×15, FR3×15,…). This schedule included a “time-out” feature that deactivated the response lever if 2 minutes elapsed without a ratio being completed. Upon reaching stable baseline responding (at least 9 weeks), chow was then introduced. Weighed amounts of laboratory chow (Laboratory Diet, 5P00 Prolab RMH 3000, Purina Mills, St. Louis, MO, USA; typically 15–20 g) were concurrently available on the floor of the chamber during the PROG sessions. At the end of the session, rats were removed from the chamber, and food intake was determined by weighing the remaining food (including spillage). Rats were trained for an additional 5 weeks so that they could they attain relatively consistent levels of baseline lever pressing and chow intake, after which drug testing began. For most baseline days rats did not receive supplemental feeding, however, over weekends and after drug tests, rats received supplemental chow in the home cage. On baseline and drug treatment days, rats normally consumed all the operant pellets that were delivered during each session.

### Experimental Procedures

For all experiments, animals received a vehicle injection 1 week prior to beginning testing in order to habituate them to being injected. All experiments used a within-group design in which each rat received all drug or vehicle treatments (IP) in their particular experiment in a randomly varied order (each treatment was received only once, one treatment per week, either on a Thursday or a Friday). Baseline training sessions (i.e., non-drug) were conducted 4 days per week.

#### Experiments 1–4: Effort-related effects of TBZ: Comparison with other drugs

#### Experiment 1: Effects of TBZ

On test days, rats (n = 14) received injections of 0.25, 0.5, 0.75, 1.0 mg/kg TBZ or vehicle, 90 minutes prior to testing.

#### Experiment 2: Effects of the DA D1 antagonist ecopipam

On test days, rats (n = 12) received injections of 0.05, 0.1, 0.2 mg/kg ecopipam or vehicle, 30 minutes prior to testing.

#### Experiment 3: Effects of the DA D2 antagonist eticlopride

On test days, rats (n = 16) received injections of 0.02, 0.04, 0.08 mg/kg eticlopride or vehicle, 30 minutes prior to testing.

#### Experiment 4: Effects of the cannabinoid CB1 antagonist and putative appetite suppressant AM4113

On test days, rats (n = 16) received injections of 4.0, 8.0, 16.0 mg/kg AM4113 or vehicle, 30 minutes prior to testing.

#### Experiments 5–8: Effort-related effects of TBZ: Reversal experiments

#### Experiment 5: Ability of the adenosine A_2A_ antagonist MSX-3 to reverse the effects of TBZ

On test days, rats (n = 8) received an injection of 0.75 mg/kg TBZ or vehicle 90 minutes prior to testing and an injection of 0.5, 1.0, 2.0 mg/kg MSX-3 or vehicle 30 minutes prior to testing.

#### Experiment 6: Ability of the catecholamine uptake inhibitor bupropion to reverse the effects of TBZ

On test days, rats (n = 10) received an injection of 0.75 mg/kg TBZ or vehicle 90 minutes prior to testing and an injection of 5.0, 10.0, 15.0 mg/kg bupropion or vehicle 30 minutes prior to testing.

#### Experiment 7: Ability of the MAO-B inhibitor deprenyl to reverse the effects of TBZ

On test days, rats (n = 10) received an injection of 0.75 mg/kg TBZ or vehicle 90 minutes prior to testing and an injection of 2.5, 5.0, 10.0 mg/kg deprenyl or vehicle 30 minutes prior to testing.

#### Experiment 8: Ability of the COMT inhibitor tolcapone to reverse the effects of TBZ

On test days, rats (n = 8) received an injection of 0.75 mg/kg TBZ or vehicle 90 minutes prior to testing and an injection of 10.0, 20.0, 30.0 mg/kg tolcapone or vehicle 60 minutes prior to testing.

### Statistical Analyses

For each experiment, total number of lever presses, highest ratio achieved, active lever time (in seconds) and gram quantity of chow consumption were analyzed with repeated measures ANOVA of data from the entire group (i.e., both high and low performers). To determine differences between treatment conditions, non-orthogonal planned comparisons using the error term from the overall ANOVA [58] was used (the number of comparisons was restricted to the number of conditions minus one). In order to assess differences between performance groups (high and low performers), each experimental group was divided in half using a median split of vehicle lever pressing for that experiment. Using this split, total number of lever presses, highest ratio achieved, active lever time and gram quantity of chow consumed were analyzed with repeated measures factorial ANOVA using performance group as the between subjects variable (Tables 1 and 2). Interaction effects and follow-up analyses are presented; a significant interaction means that the drug treatment effect differed across the performance groups.

**Table 1:** Separate analyses of high vs. low performers for experiments 1–4.

	Total Lever presses		Highest Ratio Achieved		Active Lever Time (sec)		Chow Consumption (g)	
	High	Low	High	Low	High	Low	High	Low
TBZ	#		#		#		#	
(mg/kg, IP)	β	β	β		β		β	
**VEH**	1058.57+430.98	82.14+11.92 δ	10.57+2.09	3.00+0.31 δ	887.71+159.60	301.00+46.84 δ	5.33+1.21	9.49+0.40 δ
**0.25**	614.00+261.64	88.43+30.13	7.86+1.94 *	3.29+0.57 δ	614.71+135.60 *	316.43+55.85	6.61+1.26	9.79+0.63 δ
**0.5**	337.57+206.03 *	56.43+20.77	5.57+1.46 *	2.43+0.50	416.43+80.24 *	257.86+36.60	7.49+1.01 *	9.63+0.55
**0.75**	174.43+62.98 *	46.71+12.38 *	4.71+0.75 *	2.43+0.37	367.71+38.95 *	224.57+18.48	7.76+0.58 *	9.24+0.82
**1.0**	101.00+39.91 *	30.57+7.05 *	3.29+0.61 *	2.29+0.29	282.71+46.85 *	280.86+68.10	8.41+0.48 *	9.33+0.47

Ecopipam	#		#					
(mg/kg, IP)	β		β	β				
**VEH**	1804.66+568.43	241.33+61.91	14.83+2.06	5.50+0.72 δ	1363.83+127.79	622.50+93.70	3.82+1.08	7.62+0.53
**0.05**	734.83+189.08 *	277.00+147.03	9.50+1.20 *	5.50+1.38 δ	847.17+158.25	598.50+204.56	5.90+0.50	7.13+0.54
**0.1**	444.83+161.47 *	103.33+35.28	7.00+1.41 *	3.50+0.62 δ	641.83+170.08	404.17+66.52	5.68+0.36	7.93+0.56
**0.2**	129.83+42.52 *	34.83+5.50	4.00+0.68 *	2.33+0.21 *,δ	635.00+237.75	288.17+41.70	6.87+0.57	8.73+0.57

Eticlopride	#		#		#			
(mg/kg. IP)	β	β	β	β	β			
**VEH**	1004.25+350.54	95.13+13.08 δ	10.63+1.72	3.63+0.26 δ	1151.75+204.31	352.88+82.6 δ	5.68+1.00	8.28+0.50
**0.02**	614.63+152.28	66.63+9.93 *,δ	8.63+1.05	2.88+0.30 *,δ	914.13+157.60	279.75+41.6 δ	6.23+0.62	8.29+0.43
**0.04**	287.75+81.91 *	68.63+17.20 *,δ	5.63+1.05 *	3.00+0.33 *,δ	742.50+203.45 *	313.75+70.80	6.20+0.94	8.84+0.30
**0.08**	106.63+57.02 *	38.75+4.70 *	3.13+0.80 *	2.30+0.16 *	402.13+119.83 *	223.38+26.76	7.80+0.94	8.75+0.60

AM4113	#		#					
(mg/kg, IP)	β		β					
**VEH**	452.63+187.53	86.63+10.20 δ	7.13+1.20	3.25+0.25 δ	603.88+164.70	223.63+13.53	6.30+0.94	7.53+0.20
**4**	275.13+86.00	100.75+52.03	5.50+0.94 *	3.38+0.70	535.00+144.23	266.63+47.51	4.65+0.90	4.86+0.61
**8**	183.75+64.13 *	99.38+57.10	4.63+0.84 *	3.13+0.74	595.75+126.71	264.38+87.10	3.56+0.50	4.70+0.52
**16**	129.25+52.00 *	56.63+19.51	3.63+0.70 *	2.63+0.50	622.88+136.90	268.00+42.50	2.96+0.66	3.22+0.80

# -significant performance group x drug treatment interaction,

β -significant treatment effect within performance group,

* -significantly different from VEH,

δ -significant performance group difference.

**Table 2:** Separate analyses of high vs. low performers for experiments 5–8.

	Total Lever presses		Highest Ratio Achieved		Active Lever Time (sec)		Chow Consumption (g)	
Performance	High	Low	High	Low	High	Low	High	Low
TBZ/MSX-3	#							
(mg/kg, IP)	β							
**VEH/VEH**	1960.75+385.91	298.00+114.57	16.00+1.78	5.75+1.50	1478.75+226.61	724.50+367.54	6.35+1.11	8.65+1.46
**0.75/VEH**	409.75+143.60	60.50+10.28	7.00+1.30	2.75+0.25	759.50+177.63	322.50+53.81	7.73+0.90	7.68+2.29
**0.75/0.5**	1126.00+212.71	68.75+38.83	12.00+1.08	2.75+0.75	1159.50+276.29	361.25+145.20	6.83+0.63	9.20+1.08
**0.75/1.0**	1507.50+112.28 *	317.25+228.25	14.25+0.48	5.50+2.18	1507.50+190.95	536.00+153.90	6.48+0.77	9.40+1.31
**0.75/2.0**	1587.75+496.19 *	258.75+139.26	14.00+2.20	5.25+1.31	1351.25+328.97	717.25+383.36	6.23+1.91	9.03+0.50

TBZ/BUP	#		#				#	
(mg/kg, IP)	β	β	β	β			β	β
**VEH/VEH**	750.80+168.16	157.00+61.24 δ	9.60+1.08	4.40+0.75 δ	888.00+141.17	442.80+135.78	5.82+0.47	9.68+0.55 δ
**0.75/VEH**	162.00+24.63	62.60+22.11 δ	4.60+0.40	2.60+0.40 δ	512.80+112.42	287.00+84.30	7.10+0.56	9.88+0.13 δ
**0.75/5.0**	247.60+43.36	74.60+28.68 δ	5.60+0.40	3.00+0.55 δ	692.40+255.94	294.80+90.20	6.96+0.42	9.00+0.18 δ
**0.75/10.0**	323.60+46.72	103.60+26.23 δ	6.40+0.40	3.60+0.40 *,δ	678.40+152.91	317.40+83.25	6.84+0.57	9.14+0.58 δ
**0.75/15.0**	634.20+136.36 *	173.40+68.90 *,δ	9.20+1.02 *	4.60+0.93 *,δ	789.20+113.17	433.40+134.10	4.72+0.35 *	8.42+0.60 *,δ

TBZ/DEP	#		#				#	
(mg/kg, IP)	β	β	β					B
**VEH/VEH**	734.60+144.60	93.00+16.90 δ	9.80+1.00	3.40+0.40 δ	1167.00+263.08	646.60+139.63	3.46+1.46	8.86+0.31 δ
**0.75/VEH**	58.00+14.00	42.80+8.37	3.20+0.37	2.60+0.24	344.20+52.79	400.60+133.47	7.48+0.76	8.98+0.46
**0.75/2.5**	96.60+20.53	79.00+25.60	5.20+1.46	3.80+0.73	901.00+288.41	1214.80+209.62	7.56+0.87	8.62+0.36
**0.75/5.0**	333.00+105.78 *	213.60+63.63 *	6.20+1.16 *	4.20+0.50	1131.40+300.23	641.60+107.76	5.52+1.00	8.08+0.44 δ
**0.75/10.0**	179.60+57.56	89.60+21.00	4.60+0.81	3.60+0.40	855.60+297.38	742.60+200.70	5.58+1.23	6.46+0.22 *

TBZ/TOL	#		#		#			
(mg/kg, IP)								
**VEH/VEH**	534.50+249.31	83.25+29.79 δ	8.00+1.78	3.25+0.63 δ	908.50+363.30	224.00+24.67 δ	8.00+0.70	9.83+0.50
**0.75/VEH**	56.75+6.70	35.50+12.65	2.75+0.25	2.00+0.41	282.25+46.45	213.00+39.94	8.50+0.51	9.63+0.24
**0.75/10.0**	102.75+38.50	40.00+13.31	3.50+0.65	2.25+0.49	331.75+63.34	190.75+17.51	8.60+0.27	9.53+0.21
**0.75/20.0**	152.25+69.16	48.50+14.44	4.25+0.95	2.50+0.50	451.00+176.32	203.50+22.43	8.18+0.14	9.05+0.42
**0.75/30.0**	196.50+93.29	84.75+32.91	5.00+1.08	3.00+0.82	409.50+156.22	226.50+29.02	8.43+0.65	9.33+0.39

# - significant performance group interaction, β – significant repeated measures ANOVA,

* - reversal drug significantly different from TBZ/VEH, δ – significant performance group difference.

## Results

### Experiment 1: The VMAT-2 inhibitor TBZ decreases PROG/chow performance

#### Analyses of Total Group

**Figure 1:**
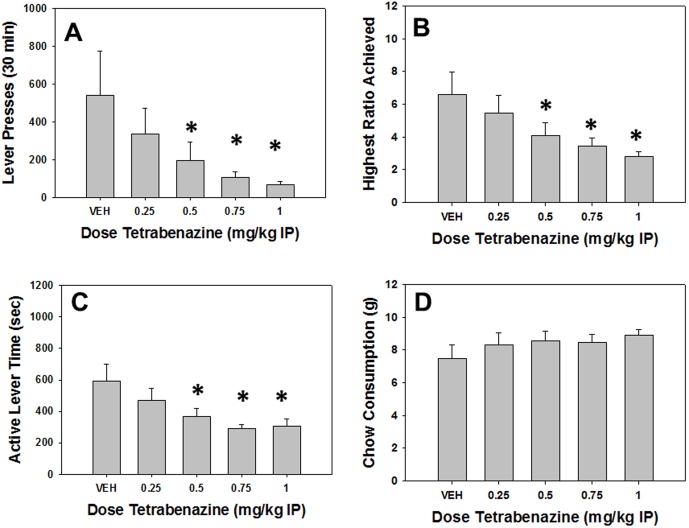
Effects of the VMAT-2 inhibitor and DA depleting agent TBZ on PROG/chow performance. On measures of lever pressing, mean (+SEM) total lever presses (A), highest ratio achieved (B), and active lever time (measured in seconds, C), TBZ produced significant decreases at 0.5, 0.75 and 1.0 mg/kg. Chow consumption (mean +SEM, in grams) during test sessions was unaffected by any dose tested (D). (* p<0.05, different from vehicle).

Repeated measures ANOVA revealed a significant effect of treatment on total lever presses (F[4], [52] = 4.204, p<0.05, Figure 1A). Planned comparisons revealed that total lever presses were significantly decreased at 0.50, 0.75 and 1.0 mg/kg TBZ compared to vehicle (p<0.05). Repeated measures ANOVA also revealed that there was a significant effect of TBZ on highest ratio achieved (F[4], [52] = 8.135, p<0.05, Figure 1B). Planned comparisons showed that highest ratio achieved was significantly decreased at 0.50, 0.75 and 1.0 mg/kg TBZ compared to vehicle (p<0.05). There also was a significant overall effect of treatment on active lever time (F[4], [52] = 6.549, p<0.05, Figure 1C), with 0.50, 0.75 and 1.0 mg/kg TBZ significantly differing from vehicle (p<0.05). Repeated measures ANOVA revealed no significant effect of TBZ on chow consumption (F[4], [52] = 2.603, n.s., Figure 1D).

#### Analyses Separating High and Low Performers

Additional analyses were performed separating high and low performers into two separate groups, and using performance group as a factor in a factorial ANOVA. With these analyses, a significant performance group x treatment interaction indicates that the drug treatment effect was different for high vs. low performers (Table 1). There was a significant performance group interaction for total lever presses (F[4], [48] = 4.242, p<0.05), highest ratio achieved (F[4], [48] = 7.378, p<0.05), active lever time (F[4], [48] = 6.458, p<0.05) and chow consumption (F[4], [48] = 4.296, p<0.05). As a result of these significant interactions, separate repeated measures ANOVAs were conducted on each performance group. In high performers, these additional analyses revealed a significant effect of TBZ on total lever presses (F[4], [24] = 4.247, p<0.05), highest ratio achieved (F[4], [24] = 10.425, p<0.05), active lever time (F[4], [24] = 9.828, p<0.05) and chow consumption(F[4], [24] = 5.000, p<0.05). Planned comparisons revealed that TBZ reduced total lever presses in high performers at 0.5, 0.75 and 1.0 mg/kg compared to vehicle (p<0.05), and also reduced highest ratio achieved and active lever time in high performers at all doses tested compared to vehicle (p<0.05), but increased chow consumption in high performers at 0.5, 0.75 and 1.0 mg/kg doses compared to vehicle (p<0.05). In contrast, repeated measures ANOVA revealed that in low performers, TBZ only affected total lever presses (F[4], [24] = 4.769, p<0.05), with planned comparisons showing that total lever presses were decreased at 0.75 and 1.0 mg/kg TBZ compared to vehicle (p<0.05). In addition, when there was a significant interaction each measure was analyzed at each treatment level for difference between the high and low performance groups. These analyses revealed that high responders lever pressed significantly more on vehicle compared to low responders (p<0.05), but the performance groups did not differ after any dose of TBZ. There also were significant performance group differences for highest ratio achieved after injection of vehicle and 0.25 mg/kg TBZ (p<0.05), but the performance groups did not differ at higher doses of TBZ. In addition, there were performance group differences in active lever time only after vehicle injections (p<0.05). For chow intake, there were significant performance group differences after injection of vehicle and 0.25 mg/kg TBZ (p<0.05), but not after higher doses of TBZ.

### Experiment 2: The DA D1 antagonist ecopipam decreases PROG/chow performance

#### Analyses of Total Group

**Figure 2:**
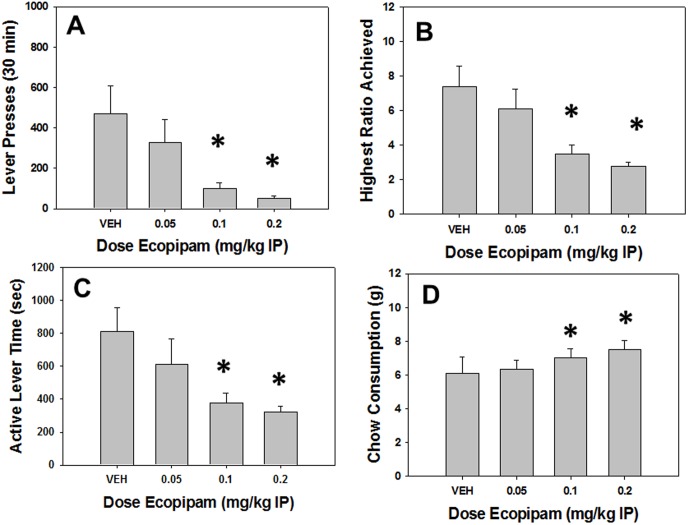
Effects of the DA D1 antagonist ecopipam on PROG/chow performance. On measures of lever pressing, mean (+SEM) total lever presses (A), highest ratio achieved (B), and active lever time (measured in seconds, C), ecopipam produced significant decreases at 0.1 and 0.2 mg/kg. Chow consumption (mean +SEM, in grams) during test sessions was unaffected by any dose tested (D). (* p<0.05, different from vehicle).

Repeated measures ANOVA revealed a significant effect of treatment on total lever presses (F[3], [33] = 6.610, p<0.05, Figure 2A). Planned comparisons showed that ecopipam significantly decreased total lever presses at 0.05, 0.1 and 0.2 mg/kg compared to vehicle (p<0.05). There was a significant effect of treatment on highest ratio achieved (F[3], [33] = 16.134, p<0.05, Figure 2B), with planned comparisons demonstrating that highest ratio achieved was significantly decreased at 0.05, 0.1 and 0.2 mg/kg ecopipam compared to vehicle (p<0.05). There also was a significant effect of treatment on active lever time (F[3], [33] = 5.667, p<0.05, Figure 2C). Active lever time was significantly decreased at 0.1 and 0.2 mg/kg ecopipam compared to vehicle (p<0.05, planned comparisons). In addition, there was a significant effect of treatment on chow consumption (F[3], [33] = 5.426, p<0.05, Figure 2D). Planned comparisons also revealed that chow consumption was significantly increased at 0.1 and 0.2 mg/kg ecopipam compared to vehicle (p<0.05).

#### Analyses Separating High and Low Performers

Repeated measures factorial ANOVA (i.e., separating animals by performance group; Table 1) revealed a significant interaction effect in total lever presses (F[3], [30] = 6.250, p<0.05), and highest ratio achieved (F[3], [30] = 7.873, p<0.05), but not for active lever time (F[3], [30] = 1.442, n.s.) or chow intake (F[3], [30] = 2.478, n.s.). Additional analyses to explore the source of the interactions were conducted as in experiment 1. In high performers, these analyses revealed a significant effect of ecopipam on total lever presses (F[3], [15] = 8.580, p<0.05) and highest ratio achieved (F[3], [15] = 26.690, p<0.05), and planned comparisons demonstrated that ecopipam decreased total lever presses and highest ratio achieved in high responders at all doses (p<0.05). In contrast, for low responders ecopipam only produced effects on highest ratio achieved (F[3], [15] = 4.153, p<0.05), leaving total lever presses not significantly affected (F[3], [15] = 2.159, n.s.). Moreover, planned comparisons revealed that ecopipam reduced highest ratio achieved in low responders only at the highest dose (p<0.05). Another source of the interaction in total lever presses was as significant difference between performance groups after vehicle treatment (p<0.05), but not at any dose of ecopipam. Moreover, analyses of the highest ratio achieved data showed that there were significant differences between high and low performers under all treatment conditions (p<0.05).

### Experiment 3: The DA D2 antagonist eticlopride decreases PROG/chow performance

#### Analyses of Total Group

**Figure 3:**
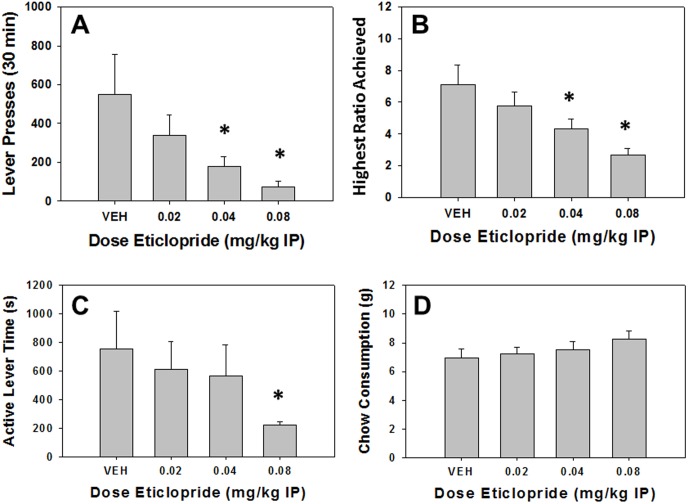
Effects of the DA D2 antagonist eticlopride on PROG/chow performance. Mean (+SEM) total lever presses (A) and highest ratio achieved (B) were significantly decreased by 0.04 and 0.08 mg/kg of eticlopride. Additionally, active lever time (measured in seconds, C) was significantly reduced at 0.08 mg/kg eticlopride. Chow consumption (mean +SEM, in grams) during test sessions was unaffected by any dose tested (D). (* p<0.05, different from vehicle).

Repeated measures ANOVA revealed a significant effect of treatment on total lever presses (F[3], [45] = 4.667, p<0.05, Figure 3A). Planned comparisons revealed that eticlopride significantly decreased total lever presses at 0.04 and 0.08 mg/kg compared to vehicle (p<0.05). There also was a significant effect of treatment on highest ratio achieved (F[3], [45] = 9.973, p<0.05, Figure 3B). Planned comparisons revealed that eticlopride significantly decreased highest ratio achieved at 0.04 and 0.08 mg/kg compared to vehicle (p<0.05). There was a significant overall treatment effect on active lever time (F[3], [45] = 6.947, p<0.05, Figure 3C). Planned comparisons also showed that active lever time was significantly decreased by eticlopride at 0.08 mg/kg compared to vehicle (p<0.05). However, there was no effect of treatment on chow consumption (F[3], [45] = 1.947, n.s., Figure 3D).

#### Analyses Separating High and Low Performers

Factorial ANOVA across performance groups (Table 1) revealed a significant interaction effect for total lever presses (F[3], [42] = 5.819, p<0.05), highest ratio achieved (F[3], [42] = 7.931, p<0.05), and active lever time (F[3], [42] = 8.640, p<0.05), but not for chow intake (F[3], [42] = 0.959, n.s.). Individual ANOVAs revealed a significant effect of eticlopride on total lever presses ([3], [21] = 5.256, p<0.05), highest ratio achieved (F[3], [21] = 11.334, p<0.05), and active lever time (F[3], [21] = 7.204, p<0.05) in high responders. Planned comparisons showed that eticlopride reduced total lever presses, highest ratio achieved and active lever time at both 0.04 and 0.08 mg/kg doses in high responders (p<0.05). In low responders, there was a significant effect of eticlopride on total lever presses (F[3], [21] = 6.547, p<0.05) and highest ratio achieved (F[3], [21] = 9.085, p<0.05) but not active lever time (F[3], [21] = 1.872, n.s.), and eticlopride significantly decreased total lever presses and highest ratio achieved at all doses (planned comparisons, p<0.05). ANOVAs at each treatment level revealed that there were significant differences between performance groups for total lever presses and highest ratio achieved after injection of vehicle, and 0.02 and 0.4 mg/kg (p<0.05), but not 0.08 mg/kg eticlopride. Moreover, there were significant performance group differences after injection of vehicle and 0.02 mg/kg eticlopride (p<0.05), but not at the 0.04 or 0.08 mg/kg doses.

### Experiment 4: The cannabinoid CB1 antagonist AM4113 decreases both lever pressing and chow consumption

#### Analyses of Total Group

**Figure 4:**
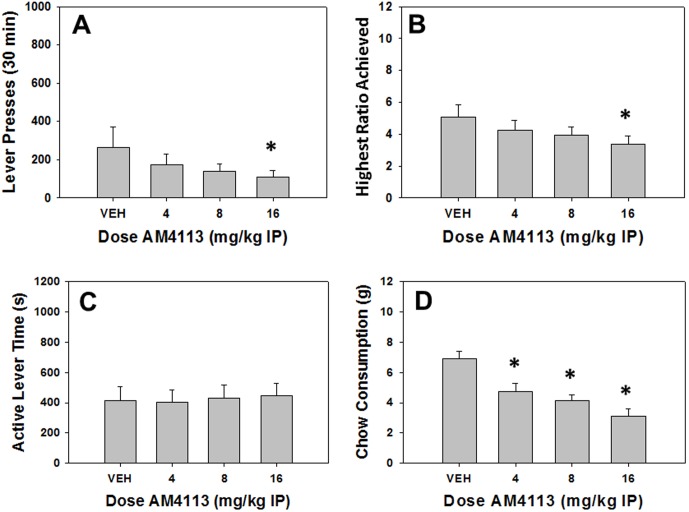
Effects of the cannabinoid CB1 antagonist and putative appetite suppressant AM4113 on PROG/chow performance. Mean (+SEM) total lever presses (A) and highest ratio achieved (B) were significantly decreased by 16.0 mg/kg of AM4113. Active lever time (measured in seconds, C) was not affected by AM4113 at any dose tested. Chow consumption (mean +SEM, in grams) was significantly reduced at 4.0, 8.0 and 16.0 mg/kg of AM4113 (D). (* p<0.05, different from vehicle).

Repeated measures ANOVA revealed a significant effect of AM4113 on total lever presses (F[3], [45] = 3.496, p<0.05, Figure 4A). Non-orthogonal planned comparisons demonstrated that AM4113 decreased total lever presses at 8.0 and 16.0 mg/kg compared to vehicle (p<0.05). There also was a significant effect of AM4113 on highest ratio achieved (F[3], [45] = 8.511, p<0.05, Figure 4B), and planned comparisons demonstrated that AM4113 decreased highest ratio achieved at 8.0 and 16.0 mg/kg compared to vehicle (p<0.05). Active lever time was not affected by AM4113 (F[3], [45] = 0.139, n.s., Figure 4C), however, there was a significant effect of AM4113 on chow consumption (F[3], [45] = 16.559, p<0.05, Figure 4D). Planned comparisons revealed that AM4113 decreased chow consumption at 8.0 and 16.0 mg/kg compared to vehicle (p<0.05).

#### Analyses Separating High and Low Performers

Factorial ANOVAs revealed significant performance group x treatment interaction effects (Table 1) for total lever presses (F[3],[42] = 3.487, p<0.05), and highest ratio achieved (F[3], [42] = 5.800, p<0.05), but no significant interaction for chow consumption (F[3], [42] = 0.458, n.s.) or active lever time (F[3], [42] = 0.200, n.s.). Repeated measures ANOVA revealed a significant effect of AM4113 on total lever presses (F[3], [21] = 4.277, p<0.05) and highest ratio achieved (F[3], [21] = 14.008, p<0.05) in high responders, and planned comparisons revealed that AM4113 significantly reduced total lever presses at 4.0 and 8.0 mg.kg doses (p<0.05) while highest ratio achieved was decreased at all doses (p<0.05) in high performers. However, there were no significant effects of AM4113 on total lever presses (F[3], [21]  =  0.580, n.s.) or highest ratio achieved (F[3], [21] = 0.948, n.s.) in low responders, which appears to be the major source of the interaction for these two variables.

### Experiment 5: MSX-3 reverses the effects of TBZ on PROG/chow performance

#### Analyses of Total Group

**Figure 5:**
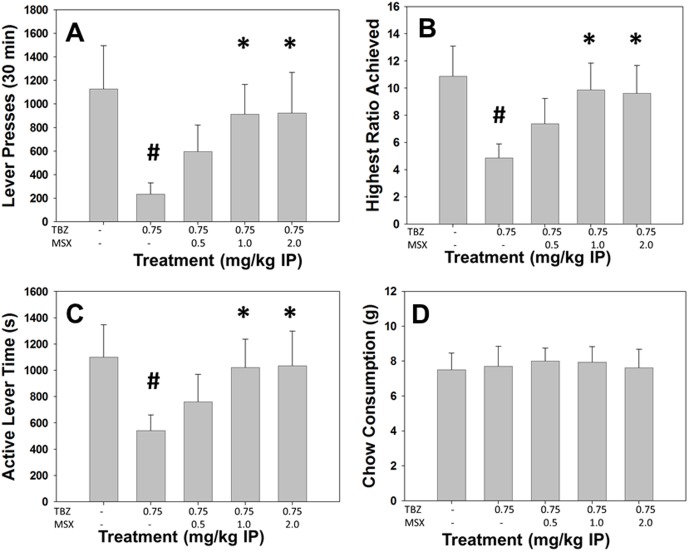
The adenosine A_2A_ antagonist MSX-3 reverses the effects of TBZ on the PROG/Chow procedure. On measures of lever pressing, mean (+SEM) total lever presses (A), highest ratio achieved (B), and active lever time (measured in seconds, C), TBZ produced significant decreases at 0.75 mg/kg. Chow consumption (mean +SEM, in grams) during test sessions was unaffected by TBZ (D). MSX-3 reversed the effects on total lever presses, highest ratio achieved and active lever time at both 1.0 and 2.0 mg/kg. (**#** p<0.05, different from vehicle; * p<0.05, different from TBZ alone).

Repeated measures ANOVA revealed a significant effect of treatment on total lever presses (F[4], [28] = 4.586, p<0.05, Figure 5A). Non-orthogonal planned comparisons revealed that TBZ significantly decreased total lever presses compared to vehicle (p<0.05), and that 1.0 and 2.0 mg/kg MSX-3 plus TBZ significantly increased total lever presses compared to TBZ alone (p<0.05). There also was a significant effect of treatment on highest ratio achieved (F[4], [28] = 6.867, p<0.05, Figure 5B). Planned comparisons revealed that TBZ significantly decreased highest ratio achieved compared to vehicle (p<0.05), and 1.0 and 2.0 mg/kg MSX-3 plus TBZ significantly increased highest ratio achieved compared to TBZ alone (p<0.05). ANOVA showed there was a significant effect of treatment on active lever time (F[4], [28] = 3.862, p<0.05, Figure 5C). TBZ significantly decreased active lever time compared to vehicle (p<0.05), and 1.0 and 2.0 mg/kg MSX-3 plus TBZ significantly differed from TBZ alone (p<0.05). There was no significant treatment effect for chow consumption (F[4], [28] = 0.102, n.s., Figure 5D).

#### Analyses Separating High and Low Performers

There were significant performance group x treatment interactions for total lever presses (F[4], [24] = 2.769, p = 0.05; Table 2), but not for highest ratio achieved (F[4], [24] = 1.765, n.s.), active lever time (F[4], [24] = 0.655, n.s.), chow consumption (F[4], [24] = 0.787, n.s.). Repeated measures ANOVA revealed a significant effect of treatment on total lever presses (F[4], [12] = 4.950, p<0.05) in high responders, and planned comparisons revealed that both 1.0 and 2.0 mg/kg doses of MSX-3 were significantly higher than TBZ alone. In contrast, repeated measures ANOVA revealed no significant effect of treatment on total lever presses (F[4], [12] = 1.051, n.s.) in low responders. High performers lever pressed at higher levels compared to low performers under all treatment conditions.

### Experiment 6: Bupropion reverses the effects of TBZ on PROG/chow performance

#### Analyses of Total Group

**Figure 6:**
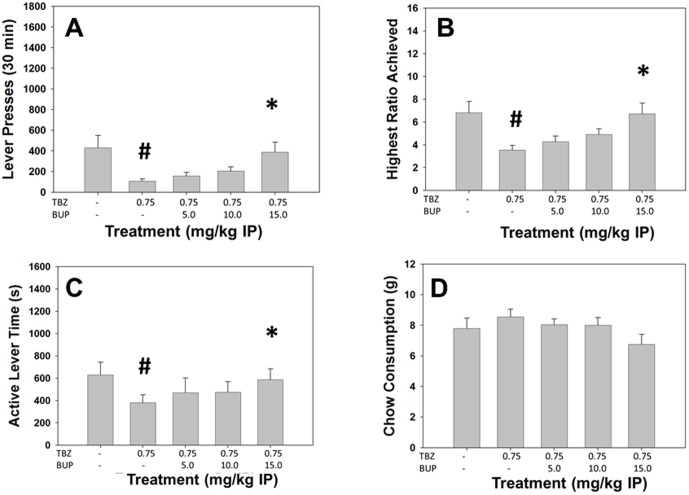
The DA uptake inhibitor and common antidepressant bupropion reverses the effects of TBZ on the PROG/Chow procedure. On measures of lever pressing, mean (+SEM) total lever presses (A), highest ratio achieved (B), and active lever time (measured in seconds, C), TBZ produced significant decreases at 0.75 mg/kg. Chow consumption (mean +SEM, in grams) during test sessions was unaffected by TBZ (D). Bupropion reversed the effects on total lever presses, highest ratio achieved and active lever time at 15.0 mg/kg. (**#** p<0.05, different from vehicle; * p<0.05, different from TBZ alone).

Repeated measures ANOVA demonstrated a significant effect of treatment on total lever presses (F[4], [40] = 7.683, p<0.05, Figure 6A). Planned comparisons revealed that total lever presses were significantly decreased by TBZ compared to vehicle (p<0.05), and that total lever presses were significantly increased at 15.0 mg/kg bupropion plus TBZ compared to TBZ alone (p<0.05). There also was a significant effect of treatment on highest ratio achieved (F[4], [40] = 14.364, p<0.05, Figure 6B). Planned comparisons showed that highest ratio achieved was significantly decreased by TBZ compared to vehicle (p<0.05), and that 15.0 mg/kg bupropion plus vehicle significantly differed from TBZ alone (p<0.05). Active lever time also showed a significant overall effect of treatment (F[4], [40] = 5.416, p<0.05, Figure 6C); this measure was significantly decreased by TBZ compared to vehicle (p<0.05), and 15.0 mg/kg bupropion plus TBZ differed from TBZ alone (p<0.05). There was a significant effect of treatment on chow consumption (F[4], [40] = 9.041, p<0.05, Figure 6D), but the only significant planned comparison was that chow intake was significantly decreased by 15.0 mg/kg bupropion compared to TBZ alone (p<0.05).

#### Analyses Separating High and Low Performers

There were significant treatment by performance group interactions (Table 2) for total lever presses (F[4], [32] = 5.730, p<0.05), and highest ratio achieved (F[4], [32] = 4.524, p<0.05), but no significant interaction for active lever time (F[4], [32] = 0.877, n.s.). Furthermore, there was a significant treatment by group interaction in chow consumption (F[4], [32] = 5.334, p<0.05). Repeated measures ANOVA revealed a significant effect of treatment on total lever presses (F[4], [16] = 7.825, p<0.05), highest ratio achieved (F[4], [16] = 10.989, p<0.05) and chow consumption (F[4], [16] = 12.029, p<0.05) in high performers, with bupropion significantly increasing total lever presses and highest ratio achieved and decreasing chow consumption at 15.0 mg/kg compared to TBZ alone (p<0.05). In low responders, there also were significant effects of treatment on total lever presses (F[4], [16] = 6.339, p<0.05), highest ratio achieved (F[4], [16] = 7.111, p<0.05) and chow consumption (F[4], [16] = 2.940, p = 0.05), and planned comparisons with this group demonstrated that bupropion significantly increased total lever presses and decreased chow consumption at 15.0 mg/kg (p<0.05), and increased highest ratio achieved at both 10.0 and 15.0 mg/kg compared to TBZ alone (p<0.05). For total lever presses, highest ratio achieved and chow intake, there were significant differences between the high and low performance groups under all treatment conditions (p<0.05).

### Experiment 7: Deprenyl reverses the effects of TBZ on PROG/chow performance

#### Analyses of Total Group

**Figure 7:**
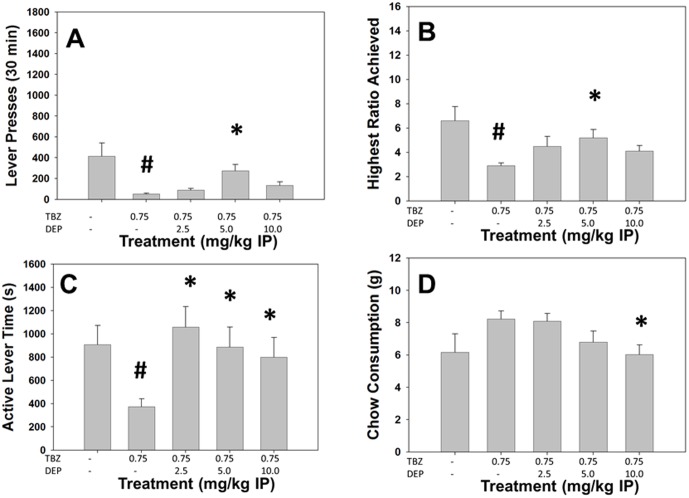
The MAO-B inhibitor and putative antidepressant deprenyl reverses the effects of TBZ on the PROG/Chow procedure. On measures of lever pressing, mean (+SEM) total lever presses (A), highest ratio achieved (B), and active lever time (measured in seconds, C), TBZ produced significant decreases at 0.75 mg/kg. Chow consumption (mean +SEM, in grams) during test sessions was unaffected by TBZ (D). Deprenyl reversed the effects on total lever presses, highest ratio achieved at 5.0 mg/kg. In addition, active lever time was increased at 2.5, 5.0 and 10.0 mg/kg. Chow consumption was significantly decreased at 10 mg/kg. (**#** p<0.05, different from vehicle; * p<0.05, different from TBZ alone).

Repeated measures ANOVA showed there was a significant effect of treatment on total lever presses (F[4], [36] = 6.172, p<0.05, Figure 7A). Planned comparisons demonstrated that total lever presses were decreased by TBZ compared to vehicle (p<0.05), and 5.0 mg/kg deprenyl plus TBZ significantly differed from TBZ alone (p<0.05). There also was a significant effect of treatment highest ratio achieved (F[4], [36] = 5.176, p<0.05, Figure 7B), with planned comparisons demonstrating that highest ratio achieved was significantly decreased by TBZ compared to vehicle (p<0.05), while 5.0 mg/kg deprenyl plus TBZ significantly differed from TBZ alone (p<0.05). Active lever time also was significantly affected by drug treatment (F[4], [36] = 3.073, p<0.05, Figure 7C). TBZ significantly suppressed active lever time compared to vehicle (p<0.05), and all doses of deprenyl plus TBZ increased active lever time compared to TBZ alone. In addition, there was a significant effect of treatment on chow consumption (F[4], [36] = 3.039, p<0.05, Figure 7D), with chow consumption being significantly lower in the 10.0 mg/kg deprenyl plus TBZ condition compared to TBZ alone (p<0.05).

#### Analyses Separating High and Low Performers

Factorial ANOVAs of each measure revealed a significant treatment x performance group interaction (Table 2) for total lever presses (F[4], [32] = 9.028, p<0.05), highest ratio achieved (F[4], [32] = 5.979, p<0.05), but not for active lever time (F[4], [32] = 1.557, n.s.). In addition, there was a significant treatment by group interaction in chow consumption (F[4], [32] = 2.911, p<0.05). In high responders, there were significant treatment effects for total lever presses (F[4], [16] = 11.097, p<0.05) and highest ratio achieved (F[4], [16] = 8.704, p<0.05), but no effect on chow consumption (F[4], [16] = 2.729, n.s.) in high responders. Planned comparisons revealed that deprenyl significantly increased total lever presses and highest ratio achieved in high responders at a dose of 5.0 mg/kg compared to TBZ alone. In the low responders, there were significant treatment effects for total lever presses (F[4], [16] = 4.677, p<0.05) and chow consumption (F[4], [16] = 7.491, p<0.05) but no effect on highest ratio achieved (F[4], [16] = 1.586, n.s.). In the low responders, planned comparisons revealed that total lever presses was significantly increased compared to TBZ alone at the 5.0 mg/kg dose of deprenyl (p<0.05), and that chow consumption was significant decreased in low responders at the dose of 10.0 mg/kg compared to TBZ alone (p<0.05). For total lever presses and highest ratio achieved, there were significant performance group differences under the vehicle condition (p<0.05), while for chow intake the performance groups differed under both the vehicle condition and the combined treatment of TBZ with 5.0 mg/kg deprenyl (p<0.05).

#### Experiment 8: Tolcapone fails to reverse the effects of TBZ on PROG/chow performanceAnalyses of Total Group

**Figure 8:**
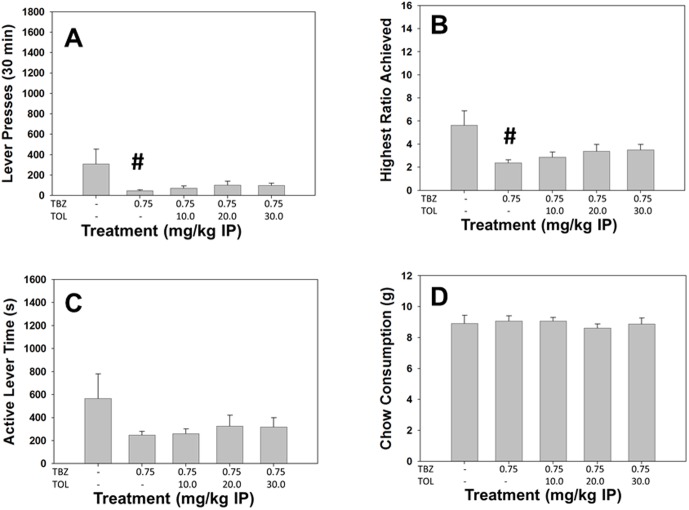
The COMT inhibitor Tolcapone fails to reverse the effects of TBZ on the PROG/Chow procedure. On measures of lever pressing, mean (+SEM) total lever presses (A), highest ratio achieved (B), and active lever time (measured in seconds, C), TBZ produced significant decreases at 0.75 mg/kg. Chow consumption (mean +SEM, in grams) during test sessions was unaffected by TBZ (D). Tolcapone did not reverse the effects on total lever presses, highest ratio achieved or active lever time at any dose tested (**#** p<0.05, different from vehicle).

Repeated measures ANOVA revealed a significant effect of treatment on total lever presses (F[4], [28] = 3.435, p<0.05, Figure 8A) and highest ratio achieved (F[4], [28] = 7.229, p<0.05, Figure 8B), but no significant effect of treatment on active lever time (F[4], [28] = 2.565, n.s., Figure 8C), or chow consumption (F[4], [28] = 0.458, n.s., Figure 8D). TBZ significantly differed from vehicle for total lever presses and highest ratio achieved, but tolcapone failed to significantly reverse any of the effects of TBZ.

#### Analyses Separating High and Low Performers

Repeated measures factorial ANOVA revealed there was a significant treatment by performance group interaction for total lever presses (F[4], [24] = 3.023, p<0.05), highest ratio achieved (F[4], [24] = 3.932, p<0.05), and active lever time (F[4], [24] = 2.904, p<0.05), but not chow consumption (F[4], [24] = 0.503, n.s.). There were no significant differences between TBZ/tolcapone and TBZ/vehicle for any of the performance groups on any measure.

## Discussion

The current studies examined the effects of the VMAT-2 inhibitor and catecholamine depleting agent TBZ on performance of the PROG/chow feeding choice task in rats. TBZ significantly decreased total lever presses, highest ratio achieved and active lever time. In addition, TBZ did not produce an overall significant effect on chow intake, indicating that appetite for chow consumption was not suppressed at the doses tested. These effects of TBZ are consistent with those recently demonstrated in rats assessed on a concurrent fixed ratio 5 (FR5)/chow feeding choice task (i.e., decreased lever pressing and increased chow intake after TBZ administration; [43]). In addition, Nunes et al. [43] reported that doses of TBZ up to 1.0 mg/kg had no effect on consumption of operant pellets or lab chow, and did not alter preference of pellets over chow, in free feeding preference tests. Thus, it seems unlikely that the present results with TBZ occurred because of a change in appetite or food preference. Importantly, these effects were more pronounced in high performers. High performers showed decreases in total lever presses at 0.5 mg/kg TBZ whereas low performers required 0.75 mg/kg in order to show decreases in total lever presses. Moreover, high performers showed decreases in highest ratio achieved and active lever time at all doses of TBZ tested, whereas low performers showed no changes in highest ratio achieved or active lever time under any dose of TBZ. Finally, high performers showed increases in chow consumption at 0.5, 0.75 and 1.0 mg/kg TBZ, where low performers showed no change in chow consumption, presumably because they were already consuming chow at or near ceiling levels.

Recent research has shown that 0.75 mg/kg TBZ significantly decreased extracellular DA in nucleus accumbens as measured by microdialysis [43]. In addition, TBZ affected expression of phosphorylated DARPP-32 in a manner consistent with reduction of DA transmission at both D1 and D2 family receptors (43). Thus, the present experiments also studied the effects of highly selective D1 and D2 family antagonists. Selective antagonism of D1 or D2 family receptors (via administration of ecopipam or eticlopride, respectively) produced similar effects to TBZ on the PROG/chow feeding task, decreasing total lever presses, highest ratio achieved and active lever time. Neither ecopipam nor eticlopride reduced chow intake in the dose range tested, and in fact, ecopipam significantly increased chow intake, which illustrates that there was a strong shift in effort-related choice from lever pressing to chow intake. Thus, it appears that reductions in DA transmission at both D1 and D2 receptors contribute to the effects induced by TBZ. Taking the present results together with previous studies, it appears that blockade of either D1 or D2 receptors, or pharmacological depletion of DA, can reduce PROG lever pressing and lower break points in a manner that does resemble the effects of appetite suppression or reinforcer devaluation [32], [43]. Instead of providing a simple measure of “reward”, PROG breakpoints represent the outcome of a cost/benefit analysis based partially upon characteristics of the reinforcer itself, but importantly, also on the work-related response costs and time constraints imposed by the increasing ratio requirements [32]. Together with earlier studies, the results of experiments 1–3 demonstrate that it is exceedingly unlikely that TBZ, ecopipam, eticlopride and haloperidol are decreasing PROG lever pressing because of a reduction in primary food motivation, appetite, or the unconditioned reinforcing properties of food [26], [32], [43]. Furthermore, these results are consistent with studies showing that increasing DA transmission by knockdown of the DA transporter [11] or overexpression of DA D2 receptors in nucleus accumbens [59] can increase instrumental response output in mice responding on fixed ratio/chow feeding choice procedures or a PROG schedule, in a manner that is inconsistent with an alteration of primary food motivation or the representation of the value of the food reinforcer.

The effects observed with TBZ or DA receptor blockade differed substantially from those induced by appetite manipulation, as demonstrated by the effects of the cannabinoid CB1 neutral antagonist and putative appetite suppressant AM4113 [35]. The effects of AM4113 described above were similar to those observed for the CB1 inverse agonist AM251 [32]; CB1 antagonism decreased chow intake as well as total lever presses and highest ratio achieved, consistent with previous studies demonstrating the appetite suppressant effects of these drugs [32], [35]. In the present studies, AM4113 produced greater effects on lever pressing in high performers compared to low performers. Specifically, high performers showed decreases in total lever presses and highest ratio achieved, whereas low performers showed no change in total lever presses. This pattern of effects in low performers is likely due to the fact that the low performing animals get the vast majority of their food from chow intake. The overall similarity between the actions of a CB1 neutral antagonist (AM4113) and a CB1 inverse agonist (AM251) suggests that there is endogenous cannabinoid tone regulating food motivated behavior, which is attenuated by occupation of CB1 receptors by the neutral antagonist [35]. Moreover, the present results highly the utility of choice procedures as compared to conventional PROG schedules, because having chow concurrently available enables one to differentiate between the effects of low doses of DA antagonists or TBZ, which leave chow consumption intact, vs. the appetite suppression induced by interference with CB1 transmission, which reduces both food reinforced lever pressing and chow intake.

In recent years, there has been increasing interest in the use of adenosine A_2A_ antagonists for their potential antidepressant effects [43], [59]. This is supported by studies using traditional animal models of depression including the forced swim and the tail suspension tests, in which adenosine A_2A_ antagonists have been shown to increase swim and struggle time [60], [61], [62]. Moreover, several studies have demonstrated the ability of A_2A_ antagonists to reverse the effects of selective DA antagonism on a variety of effort-related tasks including the FR5/chow feeding choice task and the T-maze barrier task [27], [25], [28], [39]. In the present studies, the adenosine A_2A_ antagonist MSX-3 was capable of reversing the effects of TBZ, increasing total lever presses, highest ratio achieved and active lever time. These effects are consistent with TBZ/MSX-3 reversal studies conducted in rats tested on the FR5/chow feeding choice procedure [43]. Moreover, these results are consistent with studies demonstrating the minor-stimulant properties of adenosine A_2A_ antagonists such as MSX-3, which increases operant response rates on some schedules of reinforcement [32], [57]. In the present studies, the reversal effects produced by MSX-3 were more pronounced in high performers; in these animals MSX-3 plus TBZ increased total lever presses, highest ratio achieved and active lever time relative to TBZ alone. Low performers on the other hand showed no effect of drug treatments. It is likely that this pattern of effects is due to the fact that the TBZ effects on PROG performance are greater in high performers, which could render those animals more susceptible to reversal.

In addition to testing novel compounds that are putative antidepressants, it is critical for the development and validation of the present model that the effects of well characterized antidepressants on the PROG/chow feeding choice paradigm should be assessed. Because TBZ depletes DA, a logical point of focus was on an antidepressant that works on DA, such as the catecholamine uptake inhibitor bupropion (Wellbutrin). Bupropion is one of the most widely prescribed antidepressants [63], and it is one of the few antidepressants that has shown efficacy in treating the effort-related symptoms such as fatigue, anergia and psychomotor retardation in patients with depression and related disorders [44], [46], [64]–[66]. In the current experiments, bupropion was capable of reversing the effects of TBZ on the PROG/chow procedure, increasing total lever presses, highest ratio achieved and active lever time. Moreover, at the highest dose (15.0 mg/kg) it decreased chow consumption compared to TBZ treated animals. For both high performers and low performers, bupropion increased total lever presses, highest ratio achieved and active lever time compared to TBZ/vehicle, and there also was a decrease in chow consumption in high performers. These results are consistent with previous findings on the FR5/chow choice procedure [43] and the T-maze barrier task (Yohn et al., in preparation), which also demonstrated the ability of bupropion to reverse the effects of TBZ administration.

In addition to compounds that block DA uptake such as bupropion, there has been interest in compounds that block the enzymatic breakdown of DA for their antidepressant effects. MAO inhibitors are one group of compounds that have been assessed for their antidepressant effects. Originally developed as an antiparkinsonian drug [67], the MAO-B inhibitor deprenyl has been shown to have antidepressant effects in humans [68], [69], as well as rodents tested on traditional animal models of depression, including the forced swim test and inescapable shock paradigm [70], [71]. In view of the fact that nonselective MAO inhibitors have been used as antidepressants, and deprenyl and related drugs are recommended for treating akinesia and psychomotor slowing seen in Parkinson's disease, it is reasonable to hypothesize that an MAO-B inhibitor could be useful for the treatment of psychomotor/motivational symptoms. The current studies assessed the ability of deprenyl to reverse the effects of TBZ on the PROG/chow feeding choice procedure. Deprenyl was capable of reversing these effects, increasing total lever presses, highest ratio achieved and active lever time. In addition, deprenyl decreased chow intake at the highest dose (10.0 mg/kg) compared to TBZ alone. In analyzing these results by performance level, it was revealed that these effects were greater in high performers, who demonstrated significant deprenyl-induced reversal of the effects of TBZ on total lever presses, highest ratio achieved and active lever time; low performers showed little to no effect of deprenyl. As discussed above, this could be due in part to the fact that TBZ produces greater effects in high performers, and therefore effects in these animals are easier to reverse.

COMT inhibitors are another group of drugs that act to block the enzymatic breakdown of DA, and thus could have effects on effort-related decision making. There is some clinical evidence of increased COMT levels in depressed patients [72]. Moreover, there is evidence for genetic variations in the COMT gene that could lead to abnormal COMT function [73]. The COMT inhibitor tolcapone has been used clinically in Parkinson's patients to effectively treat their effort-related depressive symptoms [45]. The current studies sought to assess tolcapone for its ability to reverse the effort-related impairments induced by TBZ on the PROG/chow task. As described above, tolcapone failed to reverse the effects of TBZ on any measure of PROG/chow performance in the dose range tested. One explanation for this lack of effect comes from clinical studies demonstrating that improvements in energy-related symptoms following tolcapone treatment in Parkinson's patients were in the presence of L-DOPA/carbidopa treatment [74]. This suggests that tolcapone may be more effective at reversing the effects of TBZ if it is co-administered with other drugs that stimulate DA transmission, such as L-DOPA or bupropion. In addition, it has been suggested that COMT plays a more important role in prefrontal cortex, where the DA transporter is sparse, compared to striatum, where the DA transporter is abundant; such a pattern has been interpreted to mean that COMT exerts a greater effect on prefrontal cortex DA metabolism, while MAO exerts a greater effects on striatal DA metabolism [75], [76]. Furthermore, there is evidence from COMT knockout mice showing that COMT has little effect, if any, on striatal DA levels [77]. When you consider these findings in the context of clinical studies demonstrating that entacapone, a peripherally restricted COMT inhibitor, also has been shown to be effective at treating depressive symptoms when co-administered with L-DOPA [78], [79], it seems possible that the antidepressant effects of COMT inhibition are due to blockade of peripheral breakdown of L-DOPA, increasing the amount that reaches the CNS. In fact, L-DOPA has been shown to improve effort-related symptoms in depressed patients [80].

In conclusion, TBZ alters effort-related choice behavior, reducing various indices of PROG lever pressing at doses that did not suppress chow intake. In fact, TBZ increased chow intake in rats with high levels of lever pressing performance. The ability of TBZ to affect effort-based decision making is consistent with research showing that other manipulations associated with depression, including stress [81], and administration of pro-inflammatory cytokines [42], which can induce fatigue-related symptoms in animals and humans [42], [82], [83], also can alter effort-based choice. The effects of TBZ on PROG output were reversed by co-administration of the adenosine A_2A_ antagonist MSX-3, as well as two drugs that have been used as antidepressants in humans (i.e., bupropion and deprenyl). Additional studies should investigate the effort-related effects of antidepressant drugs with different pharmacological profiles. However, it also should be emphasized that tests of effort-related decision making in rodents are not designed to serve as animal models of depression, in the broadest sense of the term. Rather, they are being investigated as potential models of a group of symptoms (i.e., effort-related psychomotor/motivational symptoms) that is characteristic of depression [44]–[47], [84], [85], but also is seen across multiple disorders, including conditions associated with high levels of pro-inflammatory cytokines [42], [82], [83], schizophrenia [86]–[90], and Parkinson's disease [91]. This suggestion is consistent with recent approaches in mental health research and theory that place less emphasis on traditional diagnostic categories or disorders, and instead focus on the neural circuits mediating specific pathological symptoms (i.e., the Research Domain Criteria or RDoC approach; [42], [92]).
